# Supplementary material for: Genomics data analysis via spectral shape and topology
Source: PLoS One. 2023 Apr 26;18(4):e0284820. doi: 10.1371/journal.pone.0284820 (PMC10132553; doi:10.1371/journal.pone.0284820)
Supplement: S1 File — The .pdf file includes the derivation of standardized score T3 in Eq (6) to follow the suggestion of a reviewer. (PDF) [file pone.0284820.s001.pdf]

S1: Derivation of equation (6)

The transformation in equation (6) is followed by the marginal covariance-based standardization of the multivariate mixture model proposed in [Guo M, 2014]. As discussed in Section 2.1, x is a one-dimensional vector generated from a GMM of N components and $s_j = \mathbb{1}_{\pi=j}$. Then for $s \sim \text{Multinominal}(1; c_1, \cdots, c_N)$, $x|s \sim \mathcal{N}(\sum_{j=1}^{N} s_j\mu_j, \sum_{j=1}^{N} s_j\sigma_j)$. The authors of [Guo M, 2014] show that the marginal mean and variance of x take the following forms

$$E[x] = E[E[x|s]] = \sum_{j=1}^{N} \tilde{s}_j\mu_j \tag{S1.1}$$

$$Var[x] = E_s(Var[x|s]) + Var_s(E[x|s]) = \sum_{j=1}^{N} \tilde{s}_j[\sigma_j + ((\mu_j - \sum_{j=1}^{k} \tilde{s}_j\mu_j)^2)] \tag{S1.2}$$

The corresponding standardized score T_3 is

$$T_3 = [Var(x)]^{-1/2}(x - E[x]) \qquad \text{(S1.3))}$$

Therefore, we get equation (6) by plugging in equations (S1.1) and (S1.2) into equation (S1.3)).

References

[Guo M, 2014] Guo M, Yap JT, V. d. A. A. L. N. S. A. (2014). Voxelwise single-subject analysis of imaging metabolic response to therapy in neuro-oncology. *Stat*, 3:1.
